# PHGDH Inhibits Ferroptosis and Promotes Malignant Progression by Upregulating SLC7A11 in Bladder Cancer

**DOI:** 10.7150/ijbs.74546

**Published:** 2022-08-29

**Authors:** Liliang Shen, Junfeng Zhang, Zongtai Zheng, Fuhan Yang, Shenghua Liu, Yuan Wu, Yifan Chen, Tianyuan Xu, Shiyu Mao, Yang Yan, Wei Li, Wentao Zhang, Xudong Yao

**Affiliations:** 1 Department of Urology, Shanghai Tenth People's Hospital, School of Medicine, Tongji University, 200072,Shanghai, China; 2 Urologic Cancer Institute, School of Medicine, Tongji University, Shanghai, China; 3 Department of Urology, People's Hospital affiliated to Ningbo University, Ningbo University, Ningbo, China; 4 Department of Urology, Guangdong Second Provincial General Hospital, Guangzhou, 510317, Guangdong Province, China; 5 Department of Urology, Hefei Cancer Hospital, Chinese Academy of Science, Hefei, China

**Keywords:** Bladder cancer, ferroptosis, PHGDH, SLC7A11

## Abstract

**Background:** Bladder cancer (BCa) is a prevalent urologic malignancy that shows a poor prognosis. Abnormal metabolism and its key genes play a critical role in BCa progression. In this study, the role played by PhosphoGlycerol Dehydrogenase (PHGDH), an important molecule of serine metabolism, was investigated with regard to the regulation of ferroptosis in BCa.

**Methods:** The BCa tissues of 90 patients were analyzed by RNA-sequencing for differential pathways and genes. Western blot, qPCR, and IHC were used to determine PHGDH expression in the cell lines (*in vitro*) and patient tissues (*in vivo*). R software was used to analyze PHGDH expression, prognosis, and PHGDH+SLC7A11 score. The biological functions of PHGDH were examined through organoids, and *in vitro* and *in vivo* experiments. C11 probes, electron microscopy, and ferroptosis inhibitors/ inducers were used to detect cellular ferroptosis levels. Protein profiling, co-IP, and RIP assays were used to screen proteins that might bind to PHGDH. PHGDH-targeted inhibitor NCT-502 was used to evaluate its effect on BCa cells.

**Results:** PHGDH was highly expressed in patients with BCa. Knock-down of PHGDH promoted ferroptosis, while the decreased proliferation of BCa cells. Additionally, PHGDH knock-down downregulated the expression of SLC7A11. Co-IP and mass spectrometry experiments indicate that PHGDH binds to PCBP2, an RNA-binding protein, and inhibits its ubiquitination degradation. PCBP2 in turn stabilizes SLC7A11 mRNA and increases its expression. NCT-502, a PHGDH inhibitor, promotes ferroptosis and inhibits tumor progression in BCa. The PHGDH+ SLC7A11 score was significantly correlated with patient prognosis.

**Conclusions:** To conclude, the PHGDH, via interaction with PCBP2, upregulates SLC7A11 expression. This inhibits ferroptosis and promotes the malignant progression of BCA. The results of this study indicated that NCT-502 could serve as a therapeutic strategy for BCa.

## Introduction

Bladder cancer (BCa) is the most prevailing type of malignant cancer of the urinary system, with an annual incidence of 80.5 per 100,000 and a mortality rate of 32.9 per 100,000 in China 1. Depending on the tumor invasion depth, BCa is categorized into 2 types namely, Non-Muscle Invasive BCa (NMIBC) and Muscle Invasive BCa (MIBC). The NMIBC patients showed a 31-78% recurrence rate, while 10-15% of these patients progressed to MIBC 2. The 5-year overall survival rate of MIBC patients was 40-60%. The first-line treatment for metastatic BCa is platinum-based combination chemotherapy, which clinically improves the 5-year survival rate by only 2.6-10%. Additionally, about half of the patients cannot tolerate it, and the median survival time is 15 months 3. Moreover, BCa has a high rate of incidence, recurrence, progression, and metastasis. Therefore, there is a serious need to unravel the molecular mechanisms and all therapeutic targets for the malignant progression of BCa.

Metabolic reprogramming is an important factor that promotes tumorigenesis and development. The characteristic metabolic pathways of tumor cells are expected to provide new metabolic therapeutic targets for tumor therapy 4, 5. Previous studies have determined the role of amino acid metabolism in malignant progression and immune regulation of BCa 6, 7. PHGDH (phosphoglycerate dehydrogenase) is a key enzyme for serine synthesis, which is used as a metabolic substrate to synthesize NADPH and glycine. Glycine is a raw material for the synthesis of glutathione (GSH), whereas NADPH maintains the reduced state of GSH and provides reducing power for cells 8. GSH is necessary for tumorigenesis and development. Earlier studies indicated that PHGDH played a vital role in the development of several tumors 9.

Ferroptosis is a newly discovered form of programmed cell death and results from the iron-dependent excessive oxidation of polyunsaturated fatty acids. The non-enzymatic, iron-dependent Fenton chain reaction is critical for ferroptosis 10. Three key features of ferroptosis are membrane lipid peroxidation, availability of intracellular iron, and loss of antioxidant defenses. Changes in iron, lipids, Reactive Oxygen Species (ROS), and GSH levels ​​in ferroptosis suggest that this mode of cell death is closely related to cellular metabolism 11.

More than 80% of proteins can be degraded by the Ubiquitin-Proteasome System (UPS) to regulate various cellular functions, including the degradation of misfolded, oxidized, damaged, and non-functional proteins 12 .Accumulated evidence has emphasized that UPS also plays a key role in the regulation of ferroptosis 13. Because it can selectively ubiquitinate or deubiquitinate proteins, it helps in dynamically regulating the level of intracellular iron ions. Notably, Poly(rC)-binding Protein 2 (PCBP2), an RNA-binding protein that contributes to mRNA stabilization, has also been reported to be regulated by ubiquitination 14. PCBP2 is considered to be a promoter of tumor proliferation and progression. However, whether PHGDH interacts with PCBP2 to regulate Ub levels and how this interaction regulates downstream mRNA stability and ferroptosis remains unclear.

Here, the results indicated that PHGDH can inhibit the ubiquitination of PCBP2 by interacting with PCBP2 and regulating the UPS system. PCBP2, as an RNA-binding protein, can bind and stabilize the expression of SCL7A11 mRNA, thereby inhibiting tumor ferroptosis and promoting tumor progression.

## Methods

### Data acquisition

From the BCa datasets of Shanghai Tenth People's Hospital (STPH), and the data retrieved from The Cancer Genome Atlas (TCGA, http://cancergenome.nih.gov/) and Gene Expression Omnibus (GEO, https://www.ncbi.nlm.nih.gov/geo/) databases, 90 patients were included, as they met the following inclusion criteria in this study: (1) Histologically diagnosed with BCa; (2) Available prognostic information; and (3) Available RNA expression data. In the STPH dataset, 90 BCa patients were enrolled from November 2019 to July 2021. Five BCa datasets from GEO, including GSE13507, GSE31684, GSE48075, GSE32894, and GSE48277, were included for further analysis. All studies that included human participants had to be reviewed and approved by the Ethics Committee of STPH (approval number 2021KN108), and informed consent of patients was waived owing to its retrospective nature. The protocols regarding the total RNA extraction, paired-end library generation, and RNA-sequencing in STPH were similar to those published in our earlier study 15. The Disease-Free Survival (DFS) rate of the patients in STPH was expressed as the time between post-surgery and the time that they suffered from their first recurrence or first progression, and it included metastasis or death. Tumor progression or recurrence was diagnosed according to the patients' symptoms and acquired medical images. The patients were screened and the follow-up examinations were conducted every 3-6 months after surgery via hospital visit or telephonic conversations.

### Cell lines and reagents

The cell lines, used in this study, were acquired from the Chinese Academy of Sciences (Shanghai, China). The human BCa cell lines namely, RT4, T24, UMUC3, EJ, and SW780 were used. The immortalized human normal bladder epithelial cell line, SV-HUC-1 was also used. Here, the RT4 cell line was cultured in the McCoy's 5A medium (Thermo Fisher Scientific, Inc. USA), SV-HUC-1 in the F12k medium (Sigma-Aldrich, St. Louis, MO, USA), and T24, UMUC3, EJ, and SW780 cell lines were cultured in the RPMI-1640 medium (Thermo Fisher Scientific, Inc. USA). All cell culture mediums were supplemented with 10% fetal calf serum (FCS; Thermo Fisher Scientific, Inc. USA) and 1% penicillin /streptomycin (Hyclone, Logan, UT, USA). These cell lines were incubated in an incubator containing 5% CO_2_ at 37°C. Other compounds like chloroquine (CQ, HY-17589A), necrostatin 1 (nec-1, HY-15760), erastin (HY-15763), ferrostatin-1 (fer-1, HY-100579), RSL3 (HY-100218A), NCT-502 (HY-117240), actinomycin D (HY-17559), and MG-132 (HY-13259) were purchased from MedChemExpress (Shanghai, China).

### Lipid ROS assay

The lipid ROS in the BCa cells were detected using C11-BODIPY 581/591 (RM02821, Ab clone, China), a lipid peroxidation sensor. According to the experimental design, after treating the cells with various reagents for 96 h, they were incubated with a 10 mmol/L C11-BODIPY probe at 37°C for 30 mins in the dark. All cells were washed thrice with PBS. Thereafter, C11-BODIPY (FITC, 484 nm/510 nm) could be detected using flow cytometry.

### Western blot and co-IP

The total protein in the cell lines or tissues was extracted and quantified using the Bicinchoninic Acid protein assay (BCA; Beyotime, China). Before western blotting analysis, the protein samples (40 μg each) were electrophoresed on 10% Sodium Dodecyl Sulfate-Polyacrylamide Gels (SDS-PAGE). These SDS gels were transferred to a nitrocellulose membrane (Sigma-Aldrich; Merck KGaA). These membranes were blocked with a solution containing PBS + 5% nonfat milk at room temperature for 1 hr. The membranes were then incubated with a primary antibody at 4°C, overnight. Subsequently, the membranes were washed with PBST, three times, and then incubated with horseradish peroxidase-conjugated secondary antibody for 1 hour at RT. The labeled proteins were detected and their concentration was determined by a chemiluminescent imaging system (Tanon 5200 system, Tanon, Shanghai, China).

For co-IP, lysates of 1x10^7^ BCa cells were immunoprecipitated with IP buffer containing IP antibody-coupled agarose beads, and protein-protein complexes were later subjected to Western blot. The labeled protein membranes were observed and quantified using the Tanon 5200 system. Here, IgG was used as a negative control. Table S2 displays the list of antibodies used.

### RNA isolation and qRT-PCR

The total RNA from the human cells or tissues was extracted using the TRIzol reagent (Invitrogen, Carlsbad, CA, USA), as per the kit instructions. Then a reverse transcription system kit (Vazyme Biotech Co., Ltd, China) was applied for generating the first cDNA strand. Thereafter, the qRT-PCR technique was implemented using the ChamQ Universal SYBR qPCR Master Mix (Vazyme, China) and ABI Prism 7500 sequence detection system (Applied Biosystems, CA). The qPCR parameters were as follows: 30 s at 95°C, then 40 cycles (10 s at 95°C, 30 sat 60°C). Here, GAPDH was used as the endogenous control. Then, the relative fold change was determined using the 2-ΔΔCT technique, in triplicates. Table S2 shows the list of primers used.

### Nude mice xenograft assay

For subcutaneously transplanted tumors, 1×10^7^ BCa cells were injected subcutaneously in the right armpit of 4-week-old female BALB/c nude mice. The animals were sacrificed after 4 weeks and the subcutaneous tumors were removed for subsequent experiments. Mice body weight, tumor diameter, and tumor volume were monitored once every three days. The tumor volume was determinated using the following formula: tumor volume = π/6 × length × width^2^.

### Immunohistochemistry (IHC) analysis

The tissues were fixed in cold 4% ParaFormAldehyde (PFA) and embedded into paraffin blocks. After carrying out the de-paraffinization, dehydration, antigen retrieval, and blocking processes, the tissue sections were incubated with antibodies overnight at 4°C. Then, all the tissue sections were incubated with the biotinylated goat anti-rabbit IgG for 20 mins at RT, followed by streptavidin-horseradish peroxidase for 30 mins. Finally, diaminobenzidine-H_2_O_2_ and hematoxylin were used for staining the tissues.

### Cell counting kit-8 (CCK-8) assay and the colony formation assay

In this study, the CCK-8 assay (Yeasen, Shanghai, China) was used for detecting cell proliferation. For this purpose, 2×10^3^ cells/well were seeded into 96-well plates. Then, the CCK-8 reagent (10 μl) was added to every well and all samples were incubated at 37°C for 1.5 h. Thereafter, the absorbance of the samples was measured at 450 nm using a microplate spectrophotometer (BioTek Instruments, Winooski, VT).

For colony formation assay, 1x10^3^ cells/well were seeded into 6-well plates and cultured for 14 days. After incubation, the 6-well plates were washed thrice with cold PBS, fixed with 75% ethanol, and the cells were stained with 0.1% crystal violet. The images of the stained tumor cell colonies were observed and captured using a digital camera.

### Lentivirus infection and cell transfection

The lentivirus infection was conducted to construct a stable knock-down or over-expression of PHGDH in BCa cell lines. Briefly, the plasmid was cloned into the lentivirus vector, which was further transfected into the HEK-293T cells. Then, the virus was used to infect the BCa cells. Puromycin was used to select the stable cell lines.

For cell transfection, 1x10^5^ BCa cells/well were seeded into the 6-well plates. Then, lipofectamine 3000 (Invitrogen) was used to transfect the siRNA or shRNA plasmids into the cells according to the kit's instructions. The transfection efficiency was determined using the western blotting and qPCR techniques.

### RNA immunoprecipitation (RIP) and RNA pull-down

The RIP assay was carried out using the RIP kit (BersinBio, Guangzhou, China). As per manufacturers' instructions, lysates of 1x10^7^ BCa cells were immunoprecipitated using RIP buffer containing PCBP2 antibody-coupled magnetic beads. RNA was extracted from RNA-protein complexes. Then, the expression of SLC7A11 was verified by qPCR. The results were compared to IgG, which was used as the negative control in this assay.

Furthermore, an RNA pull-down assay was carried out, where the SLC7A11-binding proteins were assessed using an RNA pull-down kit (BersinBio, Guangzhou, China). Biotin-modified SLC7A11 and Negative Control (NC) probes were incubated with total RNA overnight, followed by protein precipitation using streptavidin magnetic beads. The retrieved protein was eluted from the RNA-protein complex, and then separated and identified using western blot.

### RNA-sequencing analysis

Briefly, PHGDH over-expression cells and negative controls were assembled, and total RNA was extracted. RNA-seq was performed using Illumina HiSeq 2000 (Shanghai OE Biotech Co., Ltd.). The retrieved data was compared to the UCSC human genome (hg19) using STAR aligner v2.5, and all the hits were quantified using the feature counting software. The RNA-seq data was then analyzed using the "DESeq2" and "ClusterProfiler" software. Values with a fold change of 2 and an adjusted P-value of 0.01 were used as the cut-off values.

### Organoids

Tumor tissue was obtained by TURBT or radical cystectomy. Then, the tissue samples were cut into small pieces (1-2 mm) and the samples were digested with collagenase type IA (1 mg/ml, C9891, Sigma Aldrich) and Y-27632 (HY-10071, MCE, 10 μM) for 30 min. The cell suspension that was obtained was filtered through a 70-μm mesh and the samples were centrifuged. The pellet was resuspended in 200 μL of Basement Membrane Extract (BME, 3533-001-02, R&D) and seeded into pre-warmed 24-well plates. After BME solidified, a human bladder organoid medium was added for culture.

### Statistical analysis

The t-test, Wilcoxon test, and 1-way ANOVA were carried out to assess the association between different factors. Meta-analysis was performed using the 'metafor' R package v2.4 and 'meta' R package v4.16. SPSS v23.0 (IBM, Armonk, NY, USA) and R v3.6.1 (https://www.r-project.org/) were employed to conduct statistical analyses. For a 2-sided test, a *P-value*<0.05 was regarded as statistically significant. For survival and meta-analyses, the prognostic value of PHGDH among 33 types of tumors in the TCGA dataset was explored using the UCSCXenaShiny (https://hiplot.com.cn/advance/ucsc-xena-shiny). The Kaplan-Meier (KM) and log-rank tests were conducted to explore the prognostic value of PHGDH expression in STPH, TCGA, and GEO datasets. BCa patients were categorized into low and high PHGDH expression sets based on the optimal cut-off values in each dataset obtained by the 'survminer' R package v0.4.8. In addition, the results of univariate Cox regression analyses [including HR and 95% Confidence Interval (CI)] of six BCa datasets (TCGA, GSE48075, GSE13507, GSE31684, GSE48277, and GSE32894) were used for meta-analysis. The χ^2^-based Q-test and I^2^ statistics were carried out to estimate the heterogeneity of the meta-analysis. If the I^2^ value is >25% and the *P-value* of the Q-test is <0.05, then meta-analysis was regarded as having high heterogeneity, and the random-effect model was performed for meta-analysis. Furthermore, Begg's test was used to explore the possible publication bias, and a *P-value*>0.05 was regarded as no publication bias.

## Results

### PHGDH expression was upregulated in BCa and it was related to the BCa progression and prognosis

**Figure 1:**
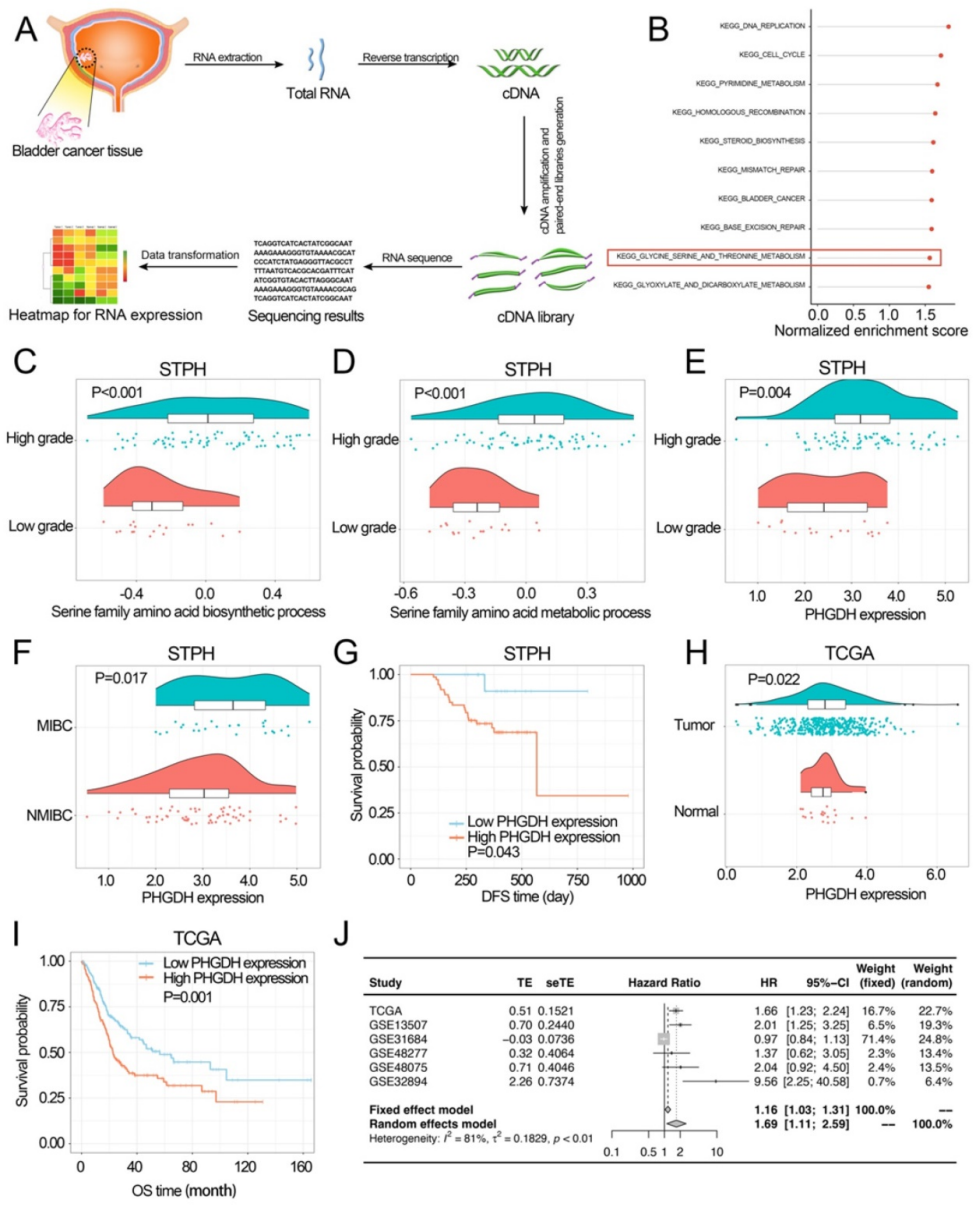
** Investigating the associations among PHGDH expression, clinicopathological properties, and prognosis of the BCa patients.** (A) The process of RNA-sequencing. (B) The top ten KEGG pathways are highly enriched in high-grade BCa of the STPH dataset. (C-D) Comparing the activities of serine family amino acid associated processes between the low-grade and high-grade BCa patients in STPH. (E) Comparing PHGDH expression between the low-grade and high-grade BCa patients in STPH. (F) Comparing PHGDH expression between NMIBC and MIBC in STPH. (G) Kaplan-Meier DFS curve for BCa patients in STPH assigned to high and low PHGDH expression groups. (H) Comparing PHGDH expression between BCa and normal tissues in TCGA. (I) KM OS curve for BCa patients in TCGA assigned to both the PHGDH expression groups. (J) Meta-analysis of HR values and its comparison in the high and the low PHGDH expression groups six BCa data sets. *p<0.05, **p<0.01, ***p<0.001.

In STPH, 90 BCa patients [73 males, 17 females; mean age = 69.5 years ± 12.1 (SD), range 31-91 years; 64 NMIBC, 26 MIBC] were enrolled for RNA-sequencing (Figure 1A). The Gene set enrichment analysis (GSEA) was carried out according to the pathological grade (Table S1). It was found that among the top 10 enriched pathways (P-value<0.05), the serine metabolism pathway was the only pathway related to amino acid metabolism. This suggests that serine metabolism disorders may promote BCa progression (Figure 1B). Further, Gene Set Variation Analysis (GSVA) revealed that serine synthesis and serine metabolism were seen to be significantly higher in high-grade BCa compared to low-grade BCa (Figure 1C-D).

In STPH, the expression of PHGDH was seen to be significantly higher in high-grade BCa than low-grade BCa and significantly higher in MIBC than NMIBC (Figure 1E-F). The analysis also revealed that patients with high PHGDH expression had shorter DFS compared to those with low PHGDH expression (Figure 1G). Next, pan-cancer survival analysis showed significant differences between various types of cancer (Figure S1 E). The expression of PHGDH in BCa and its relationship with patient prognosis in TCGA and GEO databases was further validated. Based on the data retrieved from the TCGA database, the results indicated that the PHGDH expression was significantly higher in tumor tissues than in the adjacent tissues (Figure 1H). Moreover, patients with high expression of PHGDH had a worse prognosis than those with low expression of PHGDH (Figure 1I, Figure S1 C-D, F-G). These results were validated using the GEO database (Figure S1 A-B). A meta-analysis based on six BCa datasets showed that higher PHGDH expression was related to the poor OS of BCa patients without a publication bias (Begg's test: P-value=0.573) (Figure 1J).

**Figure 2:**
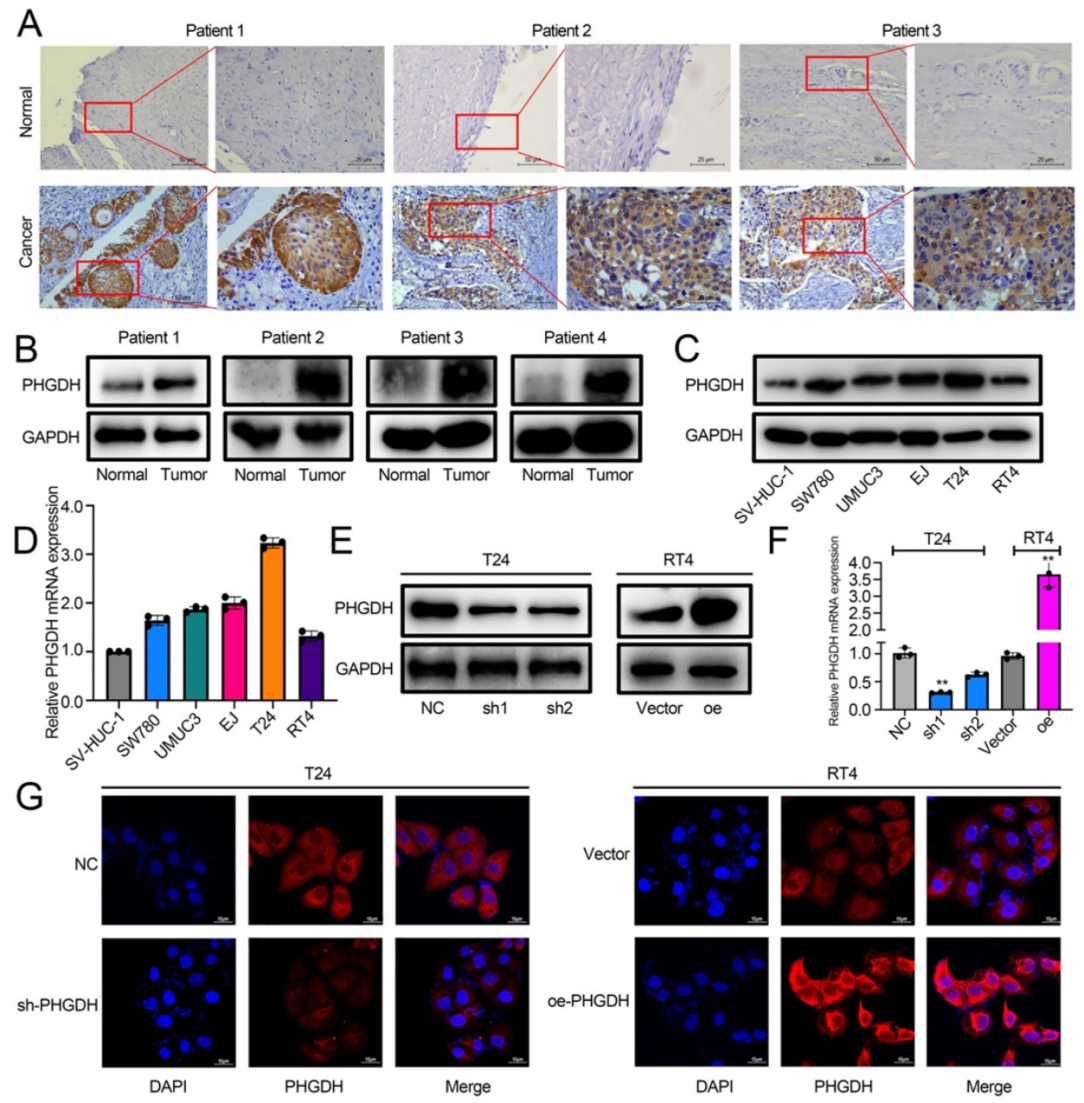
** PHGDH expression was upregulated significantly in BCa.** (A) The IHC images detailing the PHGDH expression in the tumor and normal tissues (n = 5), Scale bars: 50/25 μm. (B-C) Western blots describing the PHGDH expression in BCa tissues and cell lines. (D) A qPCR technique was used for determining the PHGDH expression in BCa cell lines. (E-F) Western blot and qPCR were used for validating the effectiveness of gene knock-down and PHGDH over-expression. (G) The immunofluorescence images for knock-down and PHGDH over-expression. Scale bars: 10 μm. *p<0.05, **p<0.01, ***p<0.001.

The role played by PHGDH in BCa was analyzed by determining its expression in cancer and adjacent tissues of BCa patients by IHC. The results showed that PHGDH was significantly over-expressed in cancer tissues of BCa patients (Figure 2A). Western blot also verified the high PHGDH expression in cancer tissues (Figure 2B). Additionally, the Western blot and qPCR results showed that the PHGDH expression was significantly higher in BCa cell lines than in normal urothelial SV-HUC-1 cell line (Figure 2C-D). Therefore, the T24 and RT4 cell lines were selected for additional experiments. A stable knock-down cell line of PHGDH was constructed in T24 and its knock-down efficiency was verified. Then, a stably transfected cell line that over-expressed PHGDH was constructed in RT4 and its over-expression efficiency was verified (Figure 2E-G).

### Knock-down and over-expression of PHGDH affect BCa proliferation and ferroptosis

**Figure 3:**
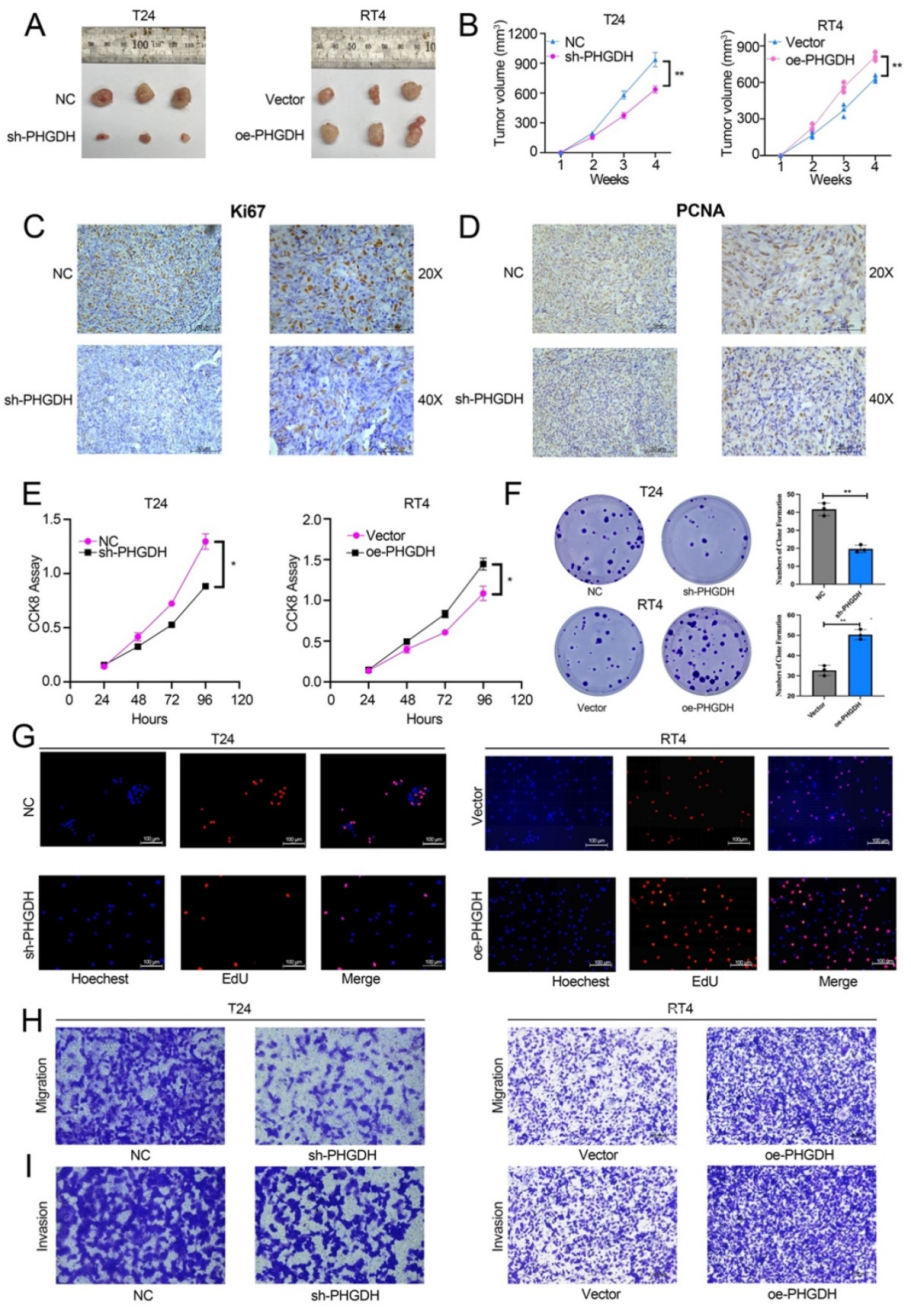
** Effect of knock-down and PHGDH over-expression on BCa proliferation and ferroptosis was verified by the *in vivo* and *in vitro* experiments.** (A-B) The subcutaneous xenograft mouse model showed that both, knock-down and over-expression of PHGDH, affect the growth of BCa *in vivo* (n=3, P-value<0.05). (C-D) Representative IHC images of Ki67 and PCNA in mouse subcutaneous tumor tissue. Scale bars: 50/25 μm. (E-F) The CCK-8 and colony formation assays were used to estimate the viability of the T24 and RT4 cell lines after PHGDH knock-down and over-expression, respectively. (G) EdU assay was used to measure the viability of T24 and RT4 cell lines after PHGDH knock-down and over-expression, respectively. (H-I) The migration and the invasion abilities of T24 and RT4 cell lines were analyzed by transwell assay after PHGDH knock-down and over-expression, respectively. *p<0.05, **p<0.01, ***p<0.001.

For determining the effect of PHGDH expression on the malignant progression of BCa, a subcutaneous tumor formation was initiated in nude mice. This subcutaneous tumor was then stripped after a month. It was found that subcutaneous tumors of mice in the PHGDH knock-down group were significantly smaller compared to that of mice in the NC group. In contrast, the subcutaneous tumors of the PHGDH over-expression group were significantly higher than that of the NC group (Figure 3A-B). IHC showed that the Ki67 and PCNA (cell proliferation markers) expression was positively related to the expression of PHGDH (Figure 3C-D, Figure S2 A-B). In addition, the CCK-8 assay confirmed that compared with the vector group, cell proliferation decreased significantly in the PHGDH knock-down group, whereas increased in the PHGDH over-expression group (Figure 3E). Clonal formation and EdU experiments also yielded the same results (Figure 3F-G). Additionally, it was noted that the ability of cell migration and invasion decreased after the knock-down of PHGDH, whereas it was enhanced after over-expression of PHGDH (Figure 3H-I).

**Figure 4:**
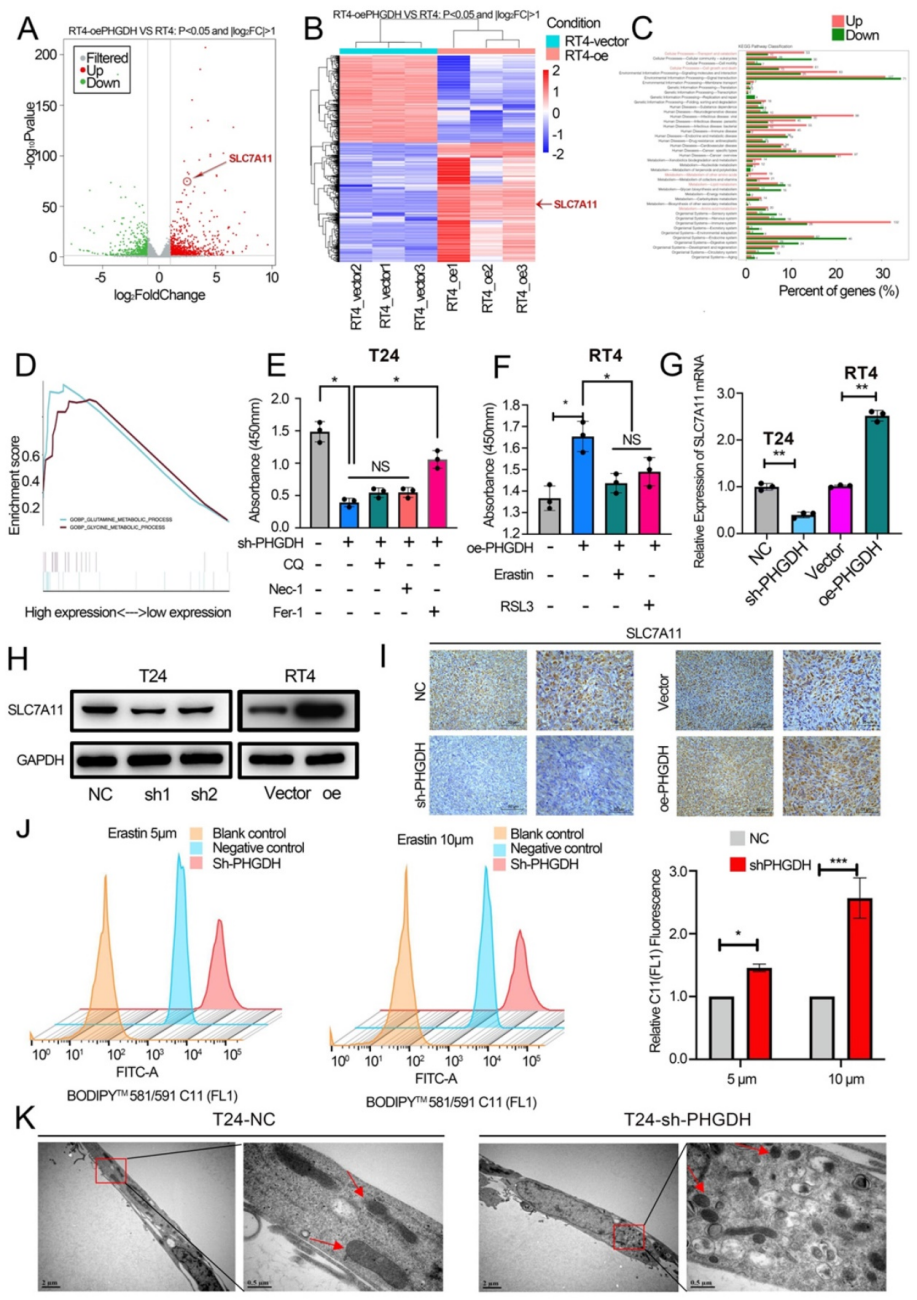
** PHGDH regulates the expression of SLC7A11 and affects cell ferroptosis.** (A-B) Volcano plots and heatmaps of RNA-sequencing. (C-D) GO enrichment analysis and GSEA of RNA-sequencing. (E) The effect on cell viability was assessed using Nec-1, CQ, and Fer-1 in the PHGDH knock-down T24 cell line. (F) Erastin and RSL3 were used to assess the effect on cell viability in the RT4 cell line over-expressing PHGDH. (G-H) Western blotting and qPCR techniques were used for verifying the SLC7A11 expression after knock-down and over-expression of PHGDH. (I) Representative IHC image of SLC7A11 in mouse subcutaneous tumor tissue. Scale bars: 50/25 μm. (J) C11 probe was used to assess lipid ROS by flow cytometry. (K) The electron microscopy technique was used for determining the changes occurring in the cellular mitochondria to assess ferroptosis. *p<0.05, **p<0.01, ***p<0.001.

Thereafter, RNA-seq was determined for the PHGDH over-expression group and vector group. Interestingly, the results showed that the expression of SLC7A11 was significantly increased (Fold change>1.5, P-value <0.05) (Figure 4A-B). GO enrichment analysis and GSEA found that over-expression of PHGDH was closely related to the ferroptosis-related pathways (including lipid metabolism, glutamine metabolism, etc.) (Figure 4C-D). It has been proved that cells die in a variety of ways, including necroptosis, apoptosis, autophagy, and ferroptosis. Here, the role played by the shPHGDH-mediated tumor inhibition in the induction of cell death was investigated. As shown in Figure 4E-F, among treatments with Nec-1, CQ, and Fer-1, which inhibit necroptosis, autophagy, and ferroptosis, respectively, only Fer-1 increases cell viability in shPHGDH cells. Moreover, Erastin and RSL3 inhibited the cell viability in RT4 cell line over-expressing PHGDH. These findings indicate that PHGDH affects cell viability through ferroptosis. Additionally, western blot and flow cytometry revealed that knock-down and over-expression of PHGDH did not affect cell apoptosis and autophagy (Figure S3 A-C). It was also noted that the mRNA and protein levels of SLC7A11 decreased significantly after PHGDH knock-down, whereas significantly increased after PHGDH over-expression (Figure 4G-H). The expression of SLC7A11 in mouse subcutaneous tumors was further verified by IHC and similar results were noted (Figure 4I). The qPCR results of 20 BCa patients indicated that the SLC7A11 expression was positively related to PHGDH expression (Figure S4 C).

The above findings were validated by assessing the lipid ROS levels. As expected, it was found that PHGDH knock-down increased the intracellular lipid ROS (Figure 4J). Furthermore, the electron microscopy analysis revealed that shPHGDH significantly induced the ferroptosis-specific changes of mitochondria, like the absence of the mitochondrial crest, mitochondrial swelling, and increase in the density of mitochondrial membrane (Figure 4K).

### PHGDH inhibitor inhibits BCa cell proliferation and promotes ferroptosis

**Figure 5:**
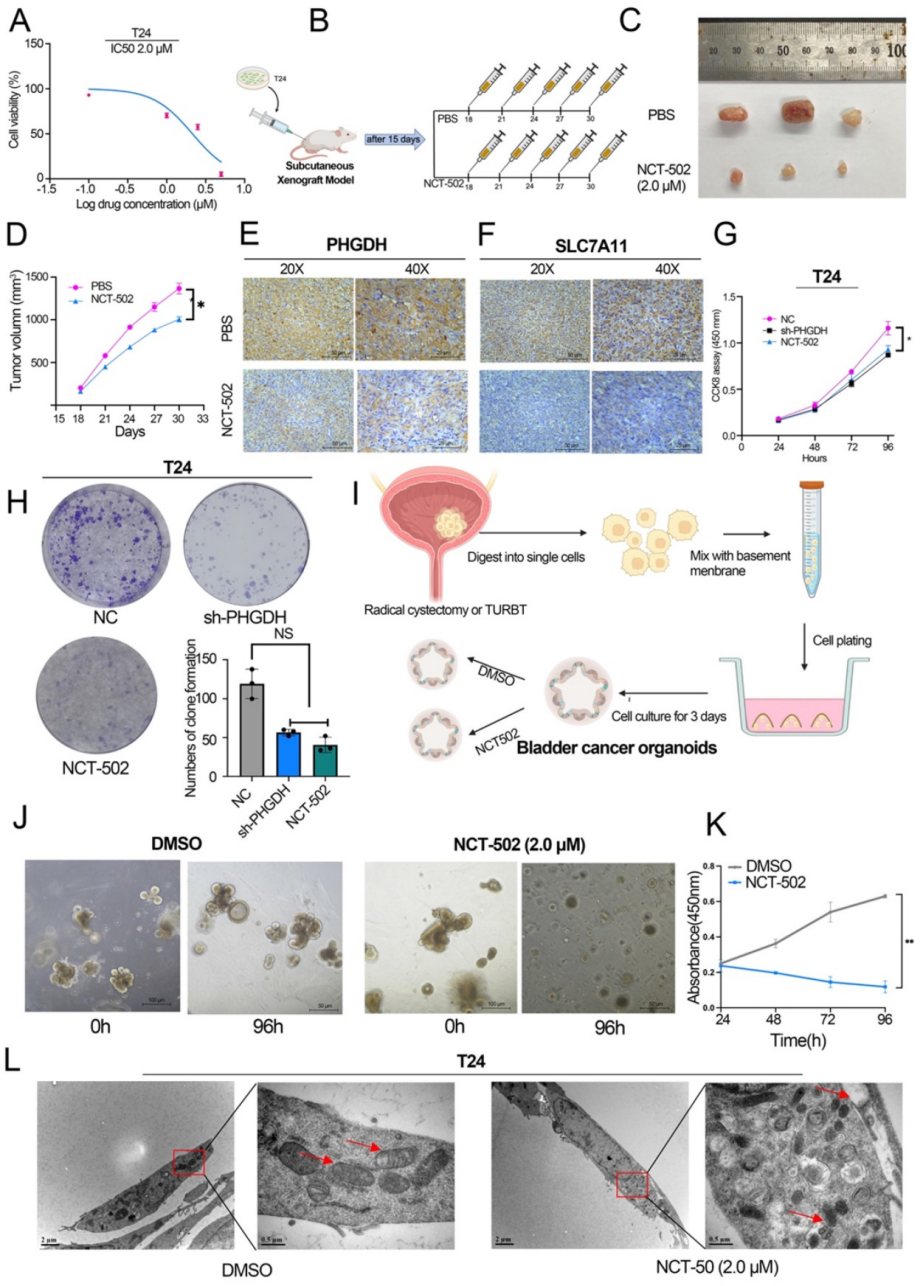
** NCT-502 inhibits BCa progression by targeting PHGDH.** (A) The IC50 value of NCT-502 in T24 cell line (B) Schematic diagram of NCT-502 xenograft model experiment (C-D) The subcutaneous xenograft mouse model showed that NCT-502 affects BCa growth *in vivo* (n=3, P-value <0.05) (E-F) Representative IHC images of PHGDH and SLC7A11 in mouse subcutaneous tumor tissues. Scale bars: 50/25 μm. (G-H) Colony formation and CCK-8 assays were used to assess cell viability after NCT-502 treatment in the T24 cell line. (I) Culture process of BCa organoids. (J-K) Effect of NCT-502 on organoid cell viability. (L) Electron microscopy technique was implemented for determining the changes in cellular mitochondria to assess ferroptosis. *p<0.05, **p<0.01, ***p<0.001.

Previous studies reveal that NCT-502, a PHGDH-specific inhibitor, inhibits the progression of various tumors 16, 17. Here, the IC50 value of NCT-502 in BCa cell lines was determined using the cell viability assay (Figure 5A, Figure S4 A). Thereafter, the influence of NCT-502 on cell proliferation of the subcutaneous xenograft model was assessed. For this, NCT-502 or vehicle control (PBS) was injected intratumorally every three days after tumor injection, and then subcutaneous xenografts were peeled off after 4 weeks. The results showed that the xenografts in the NCT-502-injected group were significantly smaller compared to those displayed by the vehicle group (Figure 5B-D). Subcutaneous tumors were then tested for PHGDH and SLC7A11 by IHC. The results indicate that expression of both, PHGDH and SLC7A11, was significantly low in the NCT-502-injected group (Figure 5E-F). Meanwhile, the impact of NCT-502 on cell proliferation was also evaluated by the CCK-8 assay. It was seen that the NCT-502-treated group showed similar cell viability as the shPHGDH group. Additionally, it was noted that the cell viability of the oePHGDH+NCT-502 group was lower when compared with the oePHGDH group (Figure 5G, Figure S4 B). Similar results were obtained for colony formation experiments (Figure 5H).

In addition, BCa organoids from 3 patients were constructed (Figure 5I). The results indicated that the cells died and lysed in the organoids co-cultured with NCT-502 for 96 h, while, the organoids cultured in DMSO grew normally (Figure 5J-K). Likewise, as with the shPHGDH results, electron microscopy analysis showed that the NCT-502 significantly induced ferroptosis-related changes in mitochondria, like the loss of mitochondrial crest, mitochondrial swelling, and increasing density of mitochondrial membrane (Figure 5L). Based on the results, it was concluded that NCT-502, a specific inhibitor of PHGDH, could be used as a potential therapeutic drug for BCa.

### PHGDH binds to PCBP2 and inhibits its ubiquitination degradation

**Figure 6:**
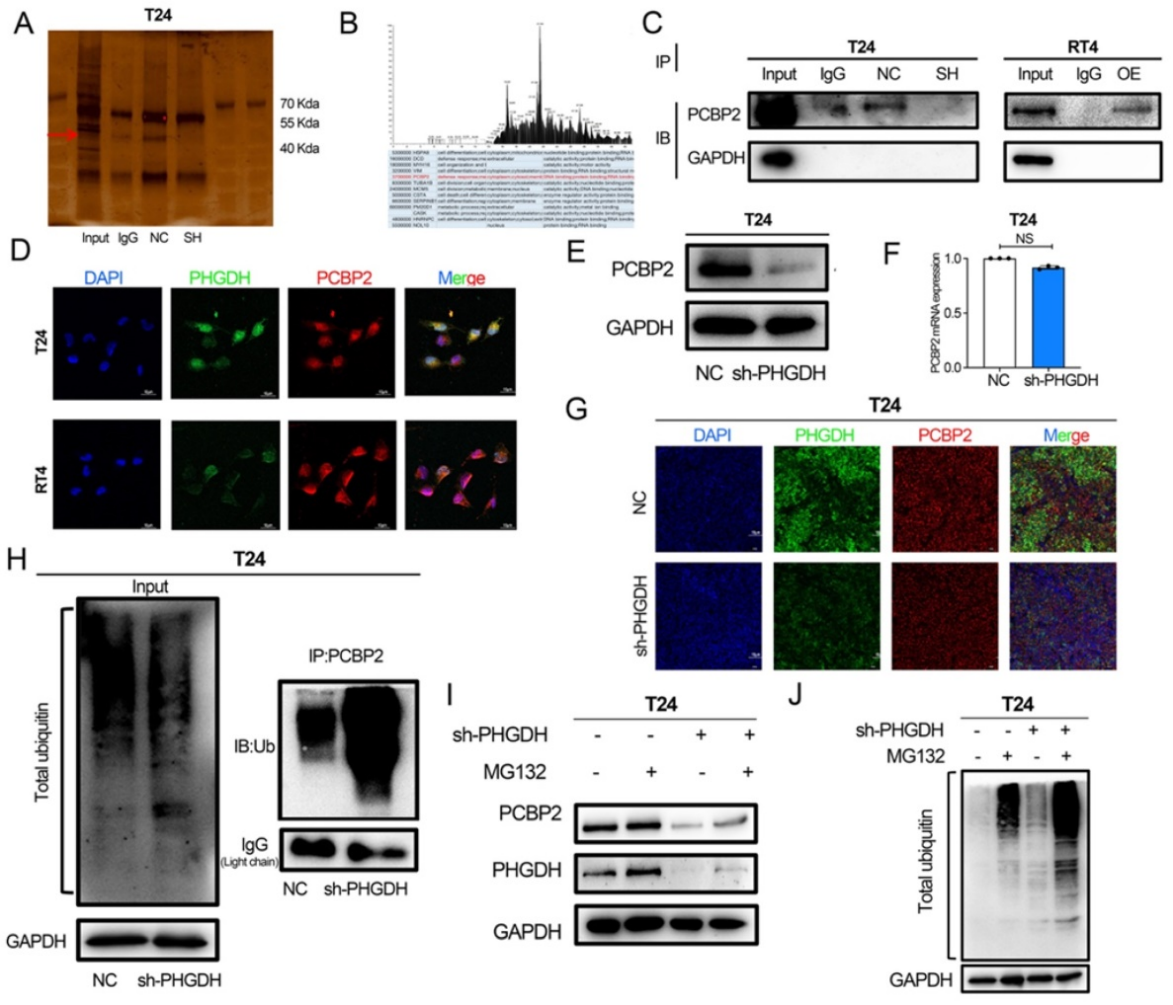
** PHGDH can bind to PCBP2 and affect its protein expression.** (A) A Co-IP assay using anti-PHGDH in T24 cells. The red arrow indicates PCBP2. (B) Peak plot of mass spectrometry (C) Western Blot after Co-IP was used to verify the binding of PHGDH with PCBP2. (D) Immunofluorescence confocal microscopy was used for determining the co-localization of PHGDH and PCBP2. (E-F) Western blot and qPCR were used to determine changes in PCBP2 after PHGDH knock-down. (G) Immunofluorescence co-staining of PHGDH and PCBP2 in mouse subcutaneous tumor tissue. (H) Co-IP experiments were used to verify PCBP2-specific ubiquitination levels. (I-J) The protein levels of PHGDH and PCBP2 and overall cellular ubiquitination levels were detected after the addition of an MG132 inhibitor. *p<0.05, **p<0.01, ***p<0.001.

Here, the process used by the PHGDH to regulate the expression of SLC7A11 mRNA was also assessed. For this purpose, the co-IP experiments were conducted and a distinct band was detected (Figure 6A) in the 40-55 protein range. Mass spectrometry was used for determining the proteins that might bind to PHGDH. The results showed that PCBP2, an RNA-binding protein, binds to PHGDH (Figure 6B). Western blot further verified that PHGDH directly binds to PCBP2 (Figure 6C). Immunofluorescence confocal microscopy of T24 and RT4 BCa cell lines confirmed the co-localization of PHGDH and PCBP2 (Figure 6D).

Furthermore, the findings revealed that the protein expression of PCBP2 was significantly reduced after the knock-down of PHGDH. However, no change was noted in the mRNA expression of PCBP2 after PHGDH knock-down (Figure 6E-F). Immunofluorescence of mouse subcutaneous tumor tissue also confirmed the down-regulation of PCBP2 protein expression after the knock-down of PHGDH (Figure 6G). The above results implied that PHGDH may regulate the PCBP2 expression through post-translational modifications. As per previous studies, knock-down of the PHGDH could activate the ubiquitin-proteasome system 18. Based on this, it was speculated that the PHGDH may affect the expression of PCBP2 through the ubiquitination pathway. This was validated by adding the proteasome inhibitor MG132 to shPHGDH. Interestingly, analysis of the Western blotting results showed that MG132 could restore the PHGDH and PCBP2 expressions (Figure 6I). Thereafter, the total ubiquitination level of the cells was determined and it was seen that the ubiquitination levels increased after the addition of MG132 (Figure 6J). Lastly, the level of PCBP2-specific ubiquitination was assessed by co-IP experiments, and the findings showed that it was significantly high in the shPCBP2 group (Figure 6H).

### PCBP2 regulates the stability of SLC7A11

**Figure 7:**
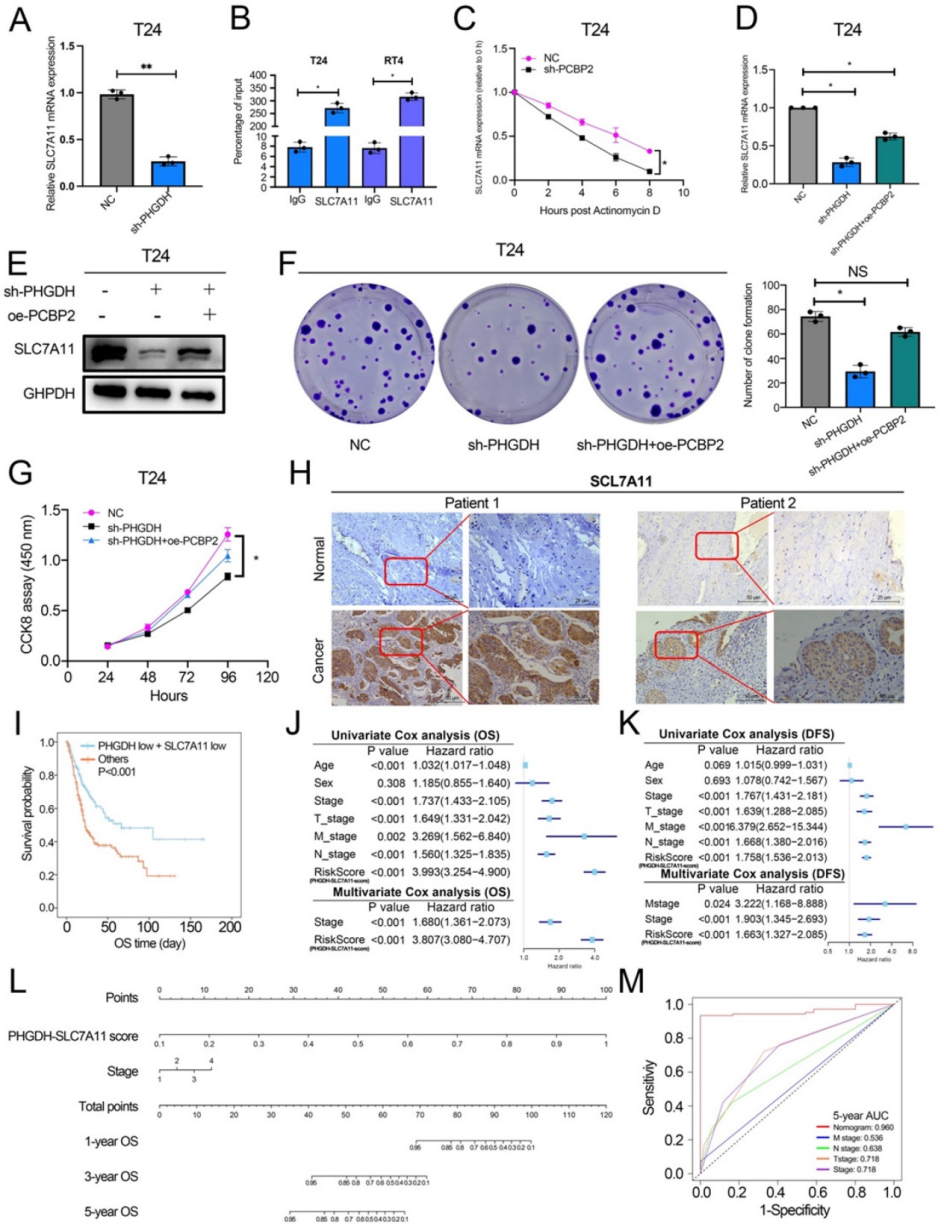
** PCBP2 stabilizes SLC7A11 mRNA and the PHGDH+SLC7A11 score can assess BCa prognosis.** (A) A qPCR technique was implemented for detecting the SLC7A11 expression after the knock-down of PCBP2. (B) RIP assay using anti-PCBP2 was used to assess the enrichment of SLC7A11 mRNA by PCBP2. IgG was regarded as the Negative control in these experiments. (C) Actinomycin D assay was used to detect the degradation of SLC7A11 mRNA. (D-E) A T24 rescue cell line was constructed by over-expressing PCBP2 in PHGDH knock-down cells, and the SLC7A11 expression was verified using the western blot and qPCR. (F-G) Colony formation and CCK-8 assays were employed for assessing the viability of the cells in the rescue cell lines. (H) Representative IHC images of SLC7A11 in patients with BCa. Scale bars: 50/25 μm. (I) KM survival analysis of patients with BCa according to PHGDH+SLC7A11 score. (J-K) Univariate and multivariate Cox regression analysis of prognostic factors for OS (J) and DFS (K). (L) A nomogram based on PHGDH+SLC7A11 score. (M) The ROC curve of nomogram and TNM staging system for the prediction of OS after 5 years. *p<0.05, **p<0.01, ***p<0.001.

Here, the mechanism used by PCBP2 for regulating SLC7A11 mRNA was also explored. The qPCR analysis revealed that the SLC7A11 expression was significantly decreased after the knock-down of PCBP2 (Figure 7A). PCBP2 is an RNA-binding protein, and RIP experiments confirmed that PCBP2 binds to the mRNA of SLC7A11 (Figure 7B). In the starBase database, PCBP2 is predicted to be significantly and positively related to the SLC7A11 expression, and the CLIP data also predicts that PCBP2 can bind to the mRNA of SLC7A11 (Figure S4D and 4G). Additionally, the RNA pull-down assay demonstrated that SLC7A11 binds to PCBP2 (Figure S4F). Meanwhile, the actinomycin D assay could be used for detecting the mRNA stability of SLC7A11. The above results showed that the mRNA stability of SLC7A11 decreased significantly after PCBP2 knock-down (Figure 7C).

Rescue experiments were used for validating the fact that the PHGDH regulates the expression of SLC7A11 through PCBP2. Western blot and qPCR revealed that over-expression of PCBP2 in shPHGDH may be partly responsible for the increased expression of SLC7A11 (Figure 7D-E). The colony formation assay showed that the shPHGDH+oePCBP2 group included a higher number of clones in comparison to the shPHGDH group (Figure 7F). The CCK-8 experiment also confirmed that cell viability was lower in the shPHGDH group than in the shPHGDH+oePCBP2 group (Figure 7G). Furthermore, the SLC7A11 expression levels in Patient 1 and Patient 2 were also assessed (as shown in Figure 2A). On a similar note, it was seen that the SLC7A11 expression was significantly higher in the patients displaying a higher PHGDH expression (Figure 7H).

Based on the above results and the TCGA database, a PHGDH+SLC7A11 score was constructed that could effectively evaluate the prognosis of patients with BCa. Accordingly, the lower the score, the better the prognosis of patients (Figure 7I, Figure S4 A-C). At the same time, the PHGDH+SLC7A11 score serves as an independent risk factor for evaluating OS and DFS in BCa patients (Figure 7J-K). Finally, a nomogram was constructed based on the PHGDH+SLC7A11 score for evaluating the prognosis of patients. The nomogram had a good predictive ability for assessing the prognosis after 3 and 5 years and the AUC value was significantly higher than the TNM staging system (Figure 7L-M, Figure S4 D-G).

## Discussion

**Figure 8:**
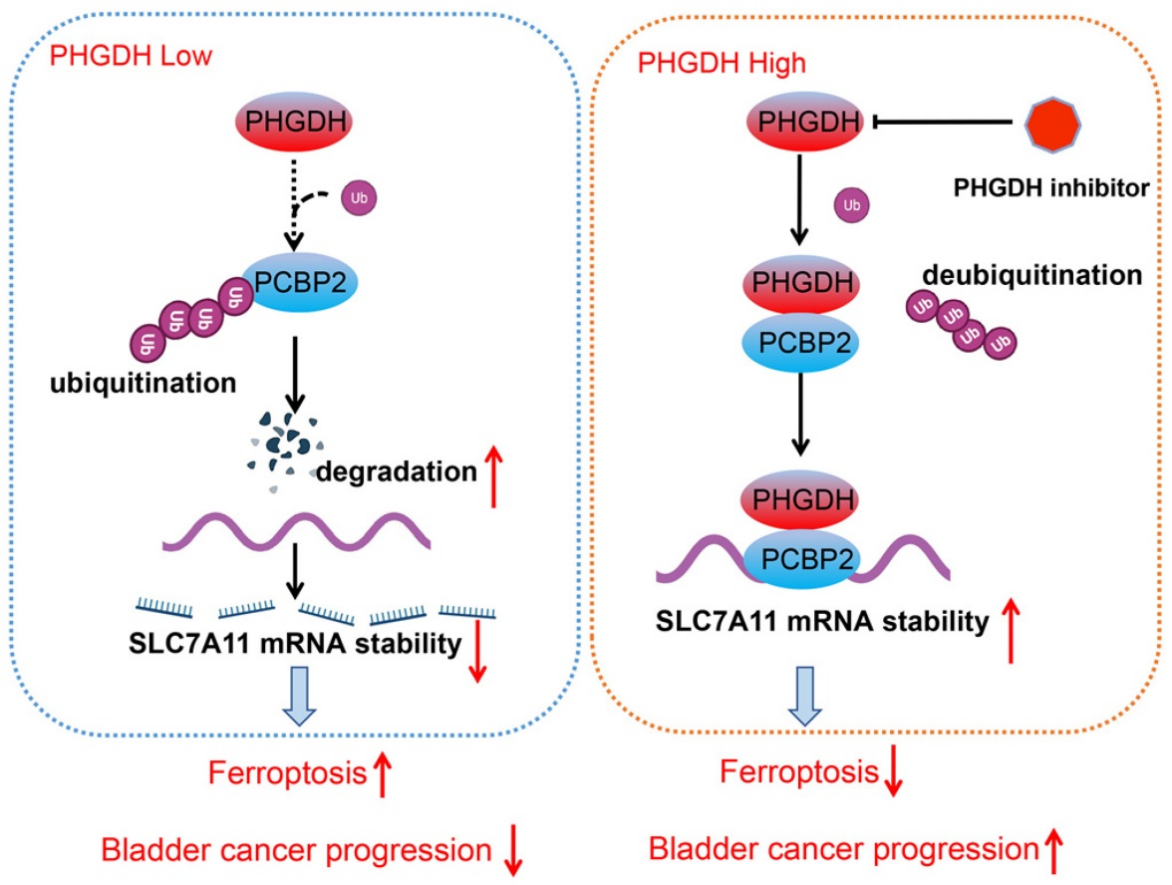
Schematic diagram depicting the mechanism by which PHGDH binds and regulates the PCBP2 expression and thereby promotes the stability of SLC7A11. This leads to inhibition of ferroptosis, and ultimately promotes tumor progression.

Metabolic reprogramming promotes the sustainable growth of tumor cells 19. Abnormal activation or dysregulation of metabolic enzymes and related pathways are considered important mechanisms that promote the malignant progression of tumors 20. Serine is a central hub of cancer metabolism, and PHGDH was seen to be the first rate-limiting step in the serine biosynthetic pathway. Recently, studies have found that PHGDH is not only important for the production of serine but also affects the occurrence and development of tumors 21.For instance, in lung cancer, high PHGDH expression is related to a poor prognosis and represents different metabolic subtypes 22. Evidence also indicates that PHGDH affected the proliferation and polarization of macrophages to regulate the tumor immune microenvironment 23. Wei et al. identified PHGDH as a key driver of sorafenib resistance in liver cancer by CRISPR/Cas9 library screening 24. Therefore, know-down of PHGDH serves to be a therapeutic target for treating cancer. Here, a novel regulatory mechanism of PHGDH was determined in BCa, which showed that PHGDH could regulate cell ferroptosis. PHGDH can bind to PCBP2 and inhibit its degradation, thereby upregulating the expression of the ferroptosis inhibitor SLC7A11, inhibiting cell ferroptosis, and leading to the malignant progression of BCa (Figure 8).

In the past few years, studies have shown that ferroptosis is involved in the onset and development of tumors. Many tumor-related genes and signaling pathways can regulate ferroptosis. Meanwhile, the unique metabolism of cancer cells, their high load of ROS, and cancer-specific mutations make some of these cells intrinsically vulnerable to iron ions 25. In urologic cancers, SUV39H1 has been found to inhibit the occurrence of cell ferroptosis and promote the progression of renal cancer 26. It has also been reported that supraphysiological doses of testosterone can induce ferroptosis in prostate cancer cells, and activate immune pathways through nuclear phagocytosis 27 . In bladder cancer, Kong et al.^28^ found that baicalin can induce ferroptosis in bladder cancer cells, and the expression of ferritin heavy chain 1 plays an important role in baicalin-induced ferroptosis. Numerous bioinformatic analysis studies have also confirmed that models constructed from ferroptosis-related molecules (mRNA and lncRNA etc.) can also predict the prognosis of bladder cancer 29, 30. Interestingly, the results in this report, showed that the overexpression of PHGDH inhibits ferroptosis, whereas PHGDH knock-down activates ferroptosis.

Solute carrier family 7 member 11 (SLC7A11) was seen to be the catalytic subunit of the amino acid transport system Xc-, which absorbed the extra-cellular cysteine molecules and reduced them to cysteine, and ultimately synthesized GSH^31^. GSH was a potent lipid peroxide scavenger and a vital cofactor for selenoenzyme Glutathione Peroxidase 4 (GPX4). It detoxifies phospholipid peroxidation and protects the cells from ferroptosis^32^. Thus, the SLC7A11-GSH system presents a major defense against ferroptosis and is the primary regulator of the cellular ferroptosis defense system 33. Earlier studies stated that the lncRNA SLC16A1-AS1 regulates the SLC7A11 expression and affects ferroptosis through sponge adsorption of miR-143-3p in renal cancer^34^. Similarly, other studies have shown that p53 can enhance ferroptosis by inhibiting SLC7A11, and this contributes to the tumor suppressor function of p53 *in vivo* and *in vitro* 35. Here, the findings confirmed that the PHGDH over-expression inhibits ferroptosis by upregulating SLC7A11.

PCBPs are a family of RNA-binding proteins and are an important form of regulatory RNA 36, 37. PCBP2 was originally identified as a component of the human α-globin mRNA complex, which can enhance mRNA stability 38. A few studies showed that PCBP2 played a vital role in several diseases, especially in the occurrence and development of cancer. PCBP2 can affect biological processes and cancer progression through RNA binding pathways. It regulates the p53 expression as it interacts with the 5'-terminal region of p53 mRNA 39. PCBP2 regulates DNA damage repair in cervical cancer as it stabilizes the RRM1 mRNA 40. The present study confirmed that PCBP2 stabilizes SLC7A11 mRNA, and that expression of SLC7A11 significantly decreased after the knock-down of PCBP2.

Out of the various protein modification processes, ubiquitination was seen to be the most common biological mechanism that affects protein stability 41, 42. Ubiquitination also plays a significant role in BCa development 43. Xie et al. observed that over-expression of TRIM65 in BCa leads to malignant progression of the tumor by ubiquitinating ANXA2^44^. Aberrantly expressed FBXW7 mediates ZMYND8 ubiquitination degradation and enhances tumor progression and stemness in BCa^45^. Previous studies found that PHGDH promotes ubiquitination of Oct4 and targets it for degradation by the proteasome. In this study, it was seen that the knock-down of PHGDH increased the mRNA expression of the proteasome subunit 18. The results of the co-IP experiments confirmed that the level of PCBP2-specific ubiquitination significantly increased after the PHGDH knock-down. In all, PHGDH inhibits the ubiquitination of PCBP2, leading to its high expression. PCBP2 in turn stabilizes SLC7A11 mRNA and increases its expression. Ultimately, increased SLC7A11 inhibits ferroptosis and promotes tumor progression.

## Conclusions

To conclude, in this report, a new mechanism used by PHGDH to regulate ferroptosis was explored. Additionally, it was noted that PHGDH inhibition serves as a potential therapeutic strategy for BCa. Furthermore, the PHGDH+SLC7A11 score helped in evaluating the prognosis of patients with BCa. However, the nomogram model constructed needs to be verified in larger samples. PHGDH knock-down also leads to changes in cell migration and invasive capacity, independent of ferroptosis. Therefore, further studies are required to explore other molecular mechanisms altered by high PHGDH in BCa.

## Supplementary Material

Supplementary figures and table legends.

Supplementary tables.

## Abbreviations

TCGA: The Cancer Genome Atlas
GEO: Gene Expression Omnibus
BCa: Bladder cancer
GSEA: Gene set enrichment analysis
PHGDH: phosphoglycerate dehydrogenase
MIBC: Muscle Invasive Bladder Cancer
NMIBC: Non-Muscle Invasive Bladder Cancer
TURBT: Transurethral Resection of Bladder Tumour
RIP: RNA immunoprecipitation
CCK-8: Cell Counting Kit 8
SLC7A11: Solute carrier family 7 member 11
PCBP2: Poly(rC)-binding Protein 2
UPS: Ubiquitin-Proteasome System
ROS: Reactive Oxygen Species
GSH: glutathione
