# Supplementary material for: PHGDH Inhibits Ferroptosis and Promotes Malignant Progression by Upregulating SLC7A11 in Bladder Cancer
Source: Int J Biol Sci. 2022 Aug 29;18(14):5459–74. doi: 10.7150/ijbs.74546 (PMC9461664; doi:10.7150/ijbs.74546)
Supplement: Supplementary file 1 — Supplementary figures and table legends. [file ijbsv18p5459s1.pdf]

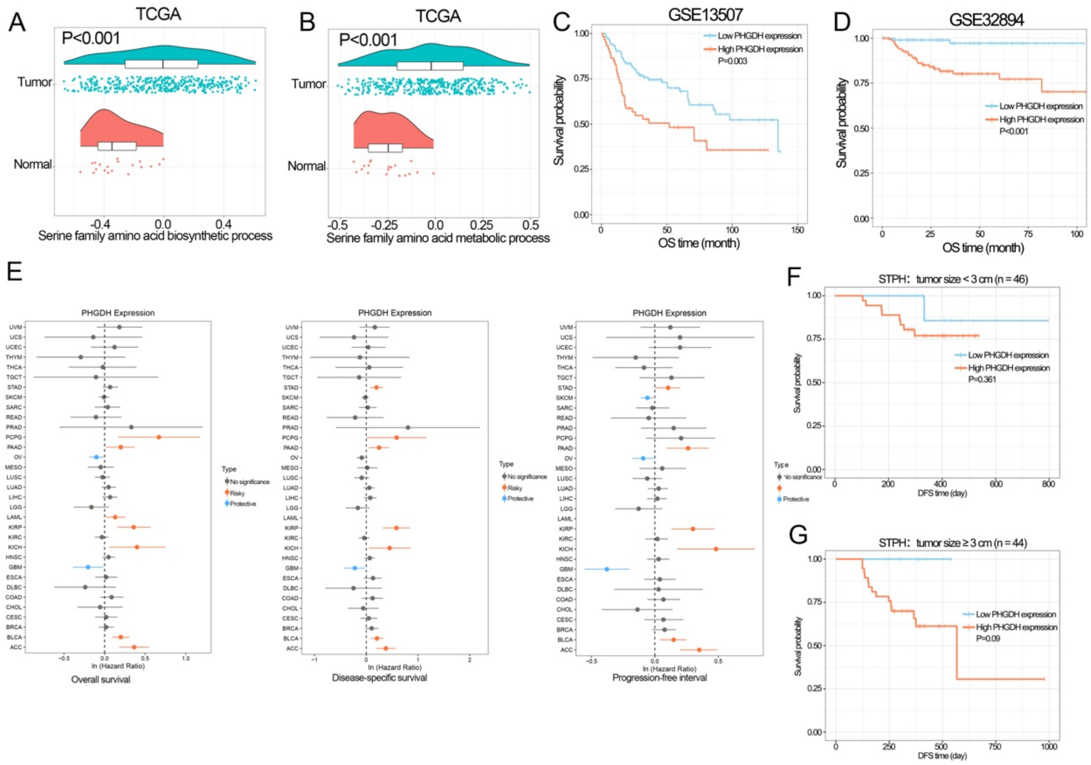

Figure S1: (A) The activities of serine family amino acid biosynthetic processes between normal and BCa cancer in TCGA. (B) The activities of serine family amino acid metabolic processes between normal and BCa cancer in TCGA. (C) Kaplan–Meier plots of PHGDH in GSE13507. (D) Kaplan–Meier plots of PHGDH in GSE32894. (E) Risk plot of correlation between PHGDH with overall survival, disease-specific survival, and progression-free interval. (F) Kaplan–Meier plots of PHGDH in BCa patient patients with tumor size < 3 cm in STPH. (G) Kaplan–Meier plots of PHGDH in BCa patient patients with tumor size ≥3 cm in STPH.

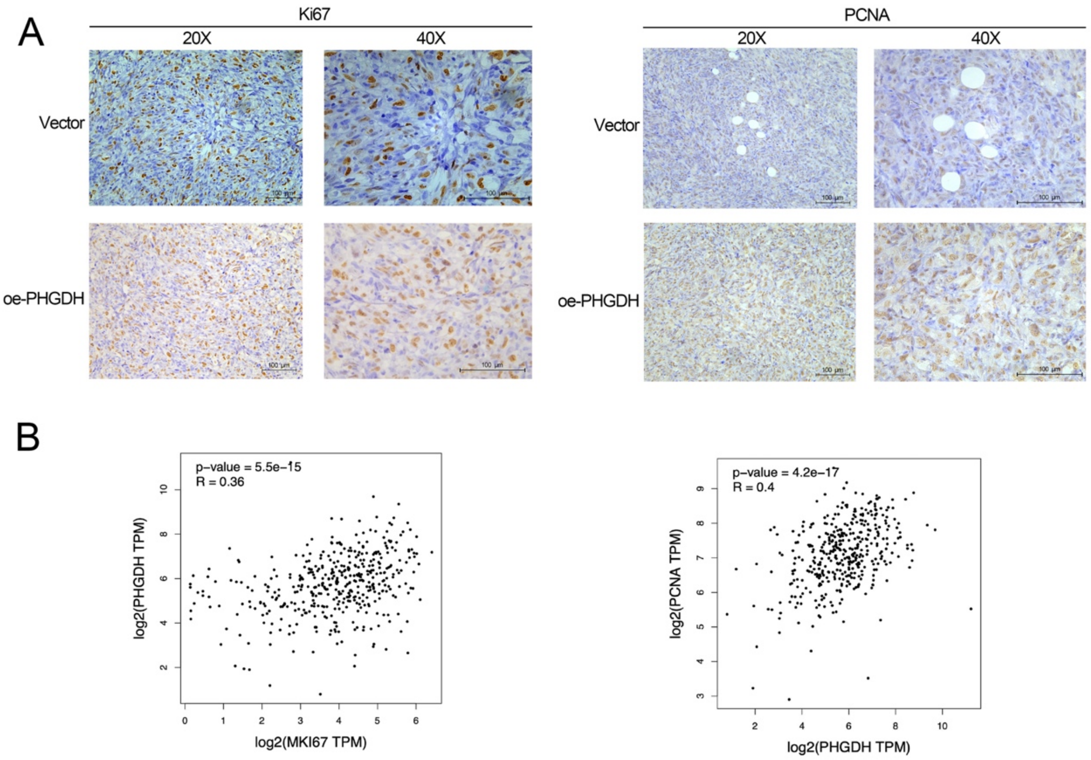

Figure S2 (A) Representative IHC images of Ki67 and PCNA in mouse subcutaneous tumor tissue (RT4 cell line), Scale bars: 50/25 μm. (B) PHGDH and Ki67/PCNA were significantly correlated based on the TCGA database.

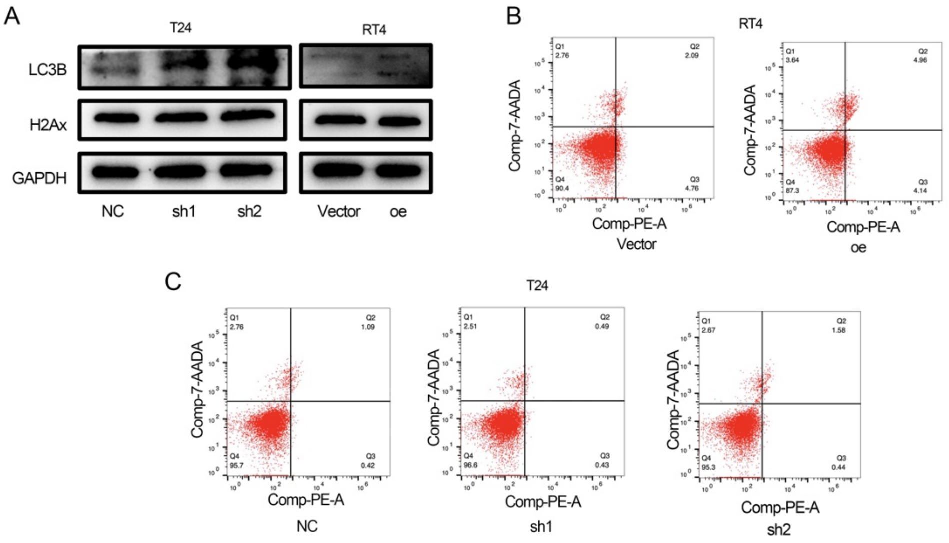

Figure S3 (A) Western blot was used to detect changes in autophagy-related proteins after knockdown or overexpression of PHGDH. (B-C) Flow cytometry was used to detect apoptosis after knockdown or overexpression of PHGDH.

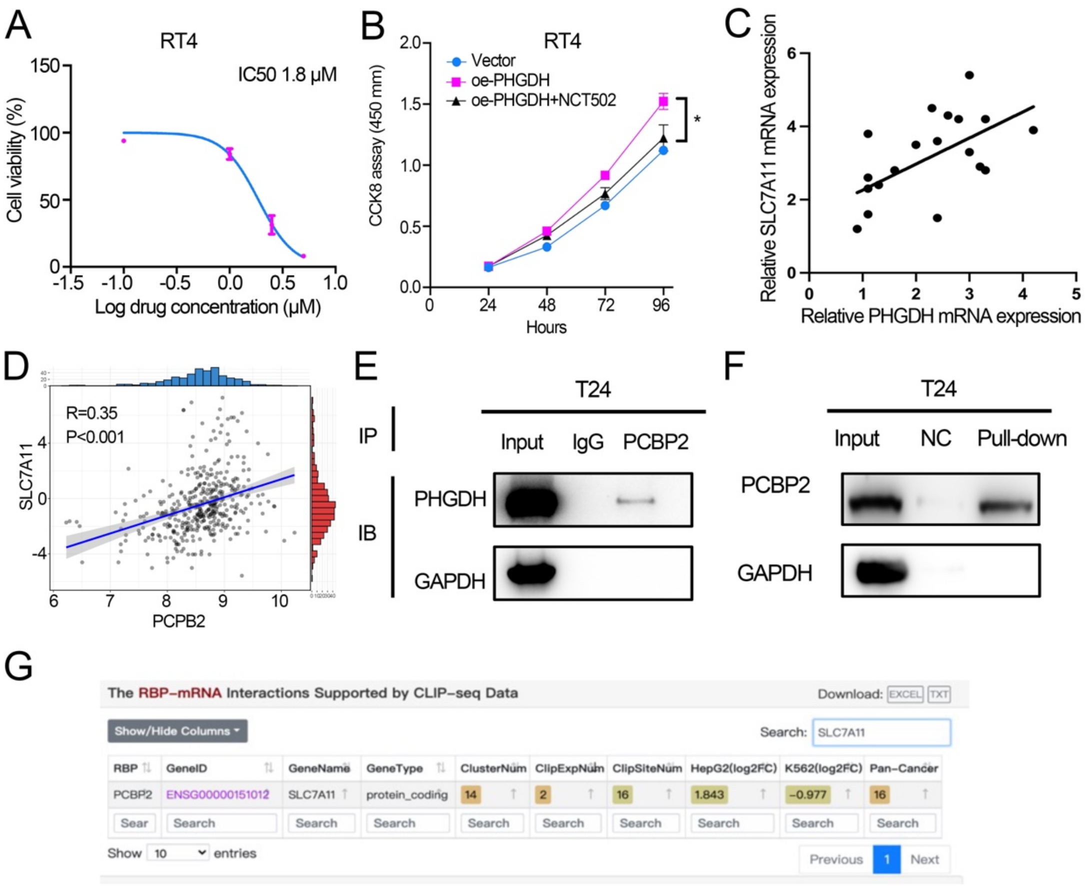

Figure S4 (A) IC50 values of NCT-502 in RT4 cell line. (B) CCK8 assays were used to validate the viability of NCT-502 in the RT4 cell line. (C) qPCR was used to detect the association of PHGDH with SLC7A11 in 20 bladder cancer patients. (D)The association of PHGDH with SLC7A11 in the TCGA database. (E) CO-IP assay was used to verify the binding of PHGDH to PCBP2 (F) RNA pull-down was used to verify the binding of PCBP2 to SLC7A11 mRNA. (G) Starbase database for prediction of PCBP2 binding to SLC7A11 mRNA.

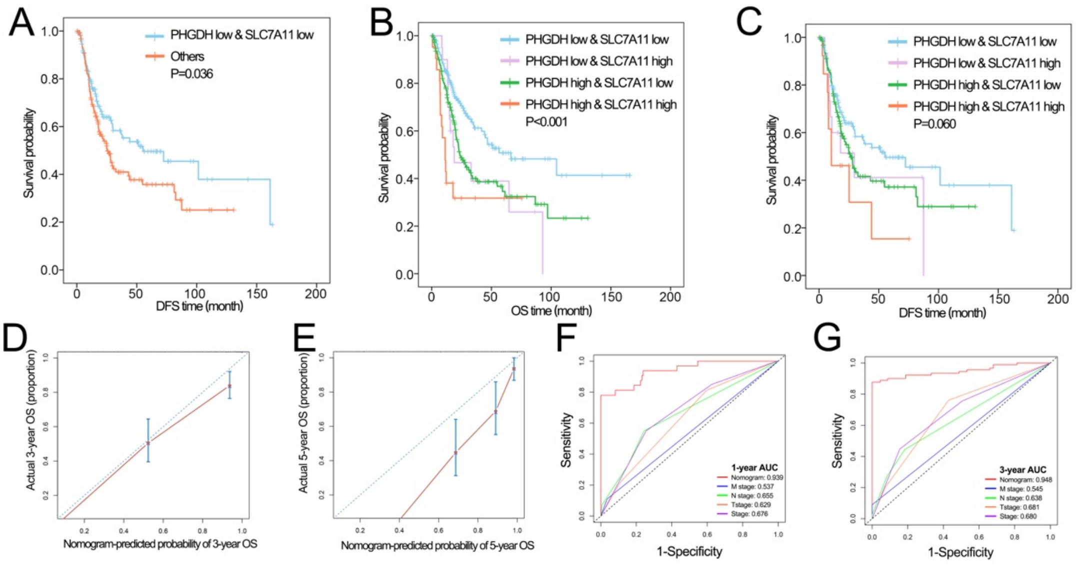

Figure S5 (A-C) Kaplan-Meier survival analysis of patients' OS and DFS according to PHGDH+SLC7A11 score. (D-E) Calibration plots of the nomogram for predicting the probability of 3- and 5-year OS. (F-G) ROC curves by nomogram compare to TNM stage for 1-(F) and 3-year OS (G).

Table S1 Clinicopathological characteristics of patients from Shanghai Tenth People's

Hospital

Table S2 List of shRNA sequence; primer and antibodies

Table S3 Data for mass spectrometry
