# Supplementary material for: PHGDH Inhibits Ferroptosis and Promotes Malignant Progression by Upregulating SLC7A11 in Bladder Cancer
Source: Int J Biol Sci. 2022 Aug 29;18(14):5459–74. doi: 10.7150/ijbs.74546 (PMC9461664; doi:10.7150/ijbs.74546)
Supplement: Supplementary file 2 — Supplementary tables. [file ijbsv18p5459s2.zip › TableS1.docx]

**Table 1** Clinicopathological characteristics of patients

|  | Number of Patients (%) | |  |
| --- | --- | --- | --- |
| Characteristic | PHGDH low (n=17) | PHGDH high  (n = 73) | P value^a^ |
| Sex |  |  |  |
| Men | 15 (88.2) | 58 (79.5) | 0.511 |
| Women | 2 (11.8) | 15 (20.5) |  |
| Age (years) |  |  |  |
| <65 | 8 (47.1) | 19 (26.0) | 0.088 |
| ≥65 | 9 (52.9) | 54 (74.0) |  |
| Tumor size (cm) |  |  |  |
| <3 | 10 (58.8) | 36 (49.3) | 0.48 |
| ≥3 | 7 (41.2) | 37 (50.7) |  |
| Number of tumors^a^ |  |  |  |
| Single | 9 (52.9) | 46 (63.0) | 0.443 |
| Multiple | 8 (47.1) | 27 (37.0) |  |
| Pathological grade |  |  |  |
| Low grade | 8 (47.1) | 10 (13.7) | 0.002* |
| High grade | 9 (52.9) | 63 (86.3) |  |
| Clinical T stage |  |  |  |
| <T2 | 15 (88.2) | 49 (67.1) | 0.136 |
| ≥T2 | 2 (11.8) | 24 (32.9) |  |
| DFS status |  |  |  |
| Event | 16 (94.1) | 51 (69.9) | 0.039* |
| Not analyzed | 1 (5.9) | 22 (30.1) |  |

DFS: disease-free survival

^a^: p value from Chi-square test

*:Statistically significant (p < 0.05)
